# Design and Electro-Thermo-Mechanical Behavior Analysis of Au/Si_3_N_4_ Bimorph Microcantilevers for Static Mode Sensing

**DOI:** 10.3390/s17112510

**Published:** 2017-11-01

**Authors:** Seok-Won Kang, Joe Fragala, Su-Ho Kim, Debjyoti Banerjee

**Affiliations:** 1 Korea Railroad Research Institute, 176 Cheoldo bangmulgwan-ro, Uiwang 16105, Gyeonggi-do, Korea; suhokim@krri.re.kr; 2 Division of Energy Environment Technology, University of Science & Technology (UST), Daejeon 34113, Korea; 3 NanoINK, Inc., 215 E Hacienda Ave., Campbell, CA 95008, USA; joefrag9@outlook.com; 4 School of Mechanical Engineering, Hanyang University, 222 Wangsimni-ro, Seongdong-gu, Seoul 04763, Korea; 5 Department of Mechanical Engineering, Texas A&M University, College Station, TX 77843, USA; dbanerjee@tamu.edu

**Keywords:** bending response, bimorph microcantilever, joule-heating, residual stress, thermal expansion coefficient (CTE)

## Abstract

This paper presents a design optimization method based on theoretical analysis and numerical calculations, using a commercial multi-physics solver (e.g., ANSYS and ESI CFD-ACE+), for a 3D continuous model, to analyze the bending characteristics of an electrically heated bimorph microcantilever. The results from the theoretical calculation and numerical analysis are compared with those measured using a CCD camera and magnification lenses for a chip level microcantilever array fabricated in this study. The bimorph microcantilevers are thermally actuated by joule heating generated by a 0.4 μm thin-film Au heater deposited on 0.6 μm Si_3_N_4_ microcantilevers. The initial deflections caused by residual stress resulting from the thermal bonding of two metallic layers with different coefficients of thermal expansion (CTEs) are additionally considered, to find the exact deflected position. The numerically calculated total deflections caused by electrical actuation show differences of 10%, on average, with experimental measurements in the operating current region (i.e., ~25 mA) to prevent deterioration by overheating. Bimorph microcantilevers are promising components for use in various MEMS (Micro-Electro-Mechanical System) sensing applications, and their deflection characteristics in static mode sensing are essential for detecting changes in thermal stress on the surface of microcantilevers.

## 1. Introduction

The development of fabrication techniques for microelectronics has facilitated the fabrication of miniaturized devices. These techniques have primarily been developed for the microfabrication of silicon-based electronic devices such as transistors, diodes, and other circuit elements. In addition, because the materials used in microelectronics, such as aluminum, silicon dioxide, silicon nitride, polycrystalline, and crystalline silicon, possess outstanding mechanical properties [1], their usage—in addition to their application of micro-metric mechanical structures (i.e., microcantilevers)—has proliferated in the field of MEMS (Micro-Electro-Mechanical Systems).

Microcantilevers have recently attracted attention as detectors in nanocalorimeters due to their high sensitivity, low analyte requirement, quick response, and so on [2,3,4,5]. This concept was pioneered based on an idea for their extensive use as the sensing platform of a probe (i.e., microcantilever) for an atomic force microscopy (AFM) [6]. In microcantilever-based MEMS sensors, monitoring mechanical deflections (i.e., static mode sensing) in thermal response to changes in temperature has frequently been adopted as the sensing mechanism. For instance, the changes in surface temperature of the microcantilever can be induced by surface catalytic reactions [2,7] or infrared (IR) absorption [8]. On the other hand, changes in the resonant frequency upon mass uptake are monitored in the dynamic mode [9]. In addition, a shift in the resonant frequency can be produced by changes in the spring constant, depending on the temperature variation [10].

Bimorph structures, which consist of a semiconductor material (e.g., silicon, silicon nitride) and a metal (e.g., aluminum, gold), have the ability to exhibit higher sensitivity in the detection of very small changes in temperature than monomaterial microcantilevers [8]. In particular, the thermal actuation caused by temperature changes relies on the mismatch in coefficients of thermal expansion (otherwise known as the bimetallic effect) between two different types of materials. An electrical resistance-based heating element spreads heat flux and causes thermal stresses. The change in deflection of electrically pre-heated microcantilevers arises from heat generation caused by adsorption of analyte species or the surface reaction.

A careful choice of the beam dimensions has to be made, in order to fabricate devices with the required resolution and sensitivity for each sensing application. In general, the overall sensitivity is determined based on the design sensitivity and the measurement sensitivity [11,12]. The Stoney equation [13] reveals the fundamentals of the surface stress-induced deflections in microcantilevers. According to the Stoney equation, larger deflections can be achieved by reducing the bending stiffness (e.g., by lowering the Young’s modulus, increasing the length, or decreasing the thickness). Thus, it is very important to determine the bending characteristics of designed microcantilevers to achieve high sensitivity of the sensing platform prior to the real fabrication of microcantilevers.

Numerical analysis is one of the most useful methods for feasibility checking and parametric studies in the process of design optimization, and can contribute to reduction of the risks, as well as the costs, of real fabrication. In general, numerical investigations have been conducted to check the implementation feasibility of the proposed mechanisms or to validate the experimental results. For instance, the dynamic behavior of the optical fiber, vibrating at its resonance frequency, was investigated by numerical analysis. The deflection of the optical fiber, modeled as a cantilever, caused by the surface stresses induced by temperature changes due to thermal actuation and amplitude variations in periodic excitation, was numerically investigated in this study [14]. Another example is the design optimization of laminated piezoresistive microcantilever sensors for static mode sensing. In this study, a numerical study was performed to determine the dimensions of the microcantilever and the doping concentration of the piezoelectric resistor for enhanced sensitivity and resolution [15]. Besides these examples, there are numerous investigations that include numerical approaches. In these studies, along with 3D numerical simulations using commercial solvers such as ANSYS, simple theoretical calculations based on commercial mathematical software such as MATLAB have successfully been made, owing to the 1D response characteristics of the microcantilevers [15,16,17,18,19,20,21,22,23,24].

The current study applies an FEA (Finite-Element Analysis) of an electric-thermal-structural coupling model to investigate the deflection characteristics of an electrically actuated bimorph microcantilever. These calculations include the modeling of the bimetallic effect induced by thermal actuation and heat generation due to electrical current, and its conductive and convective heat transfer at room temperature in air. Further, the numerically predicted deflection is compared with experimental data by optical measurement, as well as by theoretical calculation based on the model proposed in previous literature. The design optimization by theoretical calculation is devoted to enhancement of sensitivity in static mode sensing. Furthermore, the roles and importance of various factors such as dimensions, material properties, conductive/convective heat transfer characteristics at the micro-scale, and manufacturing tolerance in determination of resultant mechanical deflections due to electro-thermal actuation are discussed in this study.

## 2. Design of Bimorph Microcantilevers

### 2.1. Design Methodology

**Figure 1:**
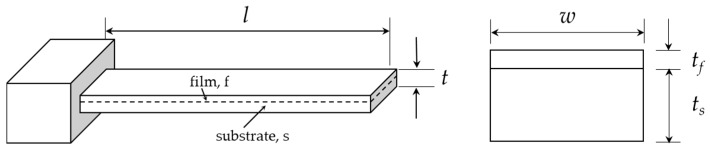
Schematic representation and cross section of simplified Si_3_N_4_/Au composite beam.

The design optimization goal for the microcantilever-based sensor utilized in static mode sensing is to find parameters that can result in larger deflections at a given surface stress. However, design parameters that obtain larger deflections can cause the degradation of the signal-to-noise ratio, due to the reduction in natural frequency. That is, any attempt to increase the deflection will decrease the resonant frequency. Accordingly, the deflection (Δ*z*) and the resonant frequency (*f_n_*) have an inverse relationship to each other, so the sensitivity is defined as Δ*z·f_n_*. In this study, the simplified bilayer structure shown in Figure 1 was considered for theoretical calculation-based sensitivity analysis (by resonance frequency and deflection contours) and optimization according to the microcantilever geometry and materials.

**Figure 2:**
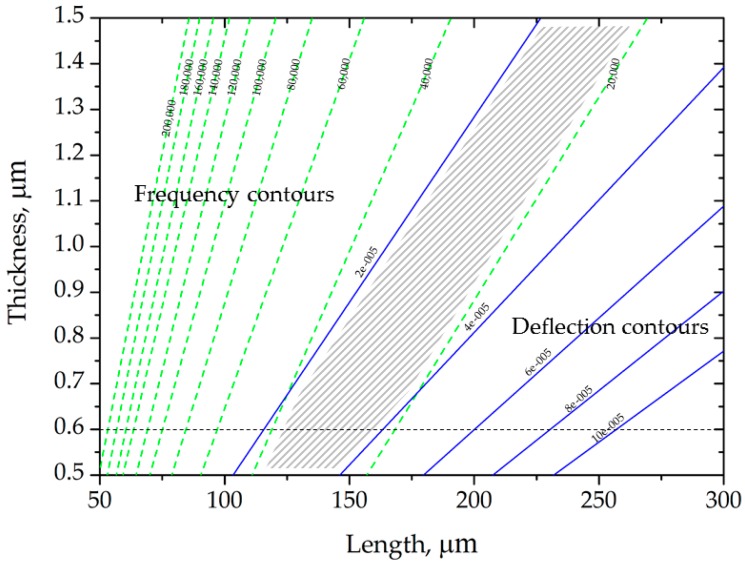
Design optimization of the simplified microcantilever for enhancing sensitivity in static mode sensing. The shaded area shows the optimum design space.

Recently, the accuracy of Stoney’s equation was improved by including higher-order terms of film thickness in solutions [17,18,19]. The curvature (*r*) and corresponding deflection for the simplified bilayer microcantilever, as given in Figure 1, due to surface stress induced by thermal actuation have also been reported in [19] as:

(1) $$\Delta z=k\frac{l^{2}}{2}\;\mathrm{where}\;k=\frac{1}{r}=\frac{6E_{s}E_{f}t_{s}t_{f}\left(t_{s}+t_{f}\right)\left(\alpha_{f}-\alpha_{s}\right)\Delta T}{E_{s}^{2}t_{s}^{4}+E_{f}^{2}t_{f}^{4}+2E_{s}E_{f}t_{s}t_{f}\left(2t_{s}^{2}+2t_{f}^{2}+3t_{s}t_{f}\right)},$$

where *l* is the length of simplified microcantilever, *E* is Young’s modulus, *t* is the thickness, *β* is the coefficient of thermal expansion, and the subscripts *s* and *f* denote the substrate and the film, respectively. Further, the resonant frequency for a rectangular profile microcantilever was given by

(2a) $$f_{n}=\frac{1}{2\pi }\cdot \sqrt{\frac{E_{eff}}{\rho_{eff}}}\cdot \frac{t}{l^{2}},$$

where

(2b) $$E_{eff}=\frac{E_{f}^{2}{\left(\frac{t_{f}}{t}\right)}^{4}+E_{s}^{2}{\left(\frac{t_{s}}{t}\right)}^{4}+2E_{f}E_{s}\frac{t_{f}}{t}\frac{t_{s}}{t}\left[2{\left(\frac{t_{f}}{t}\right)}^{2}+2{\left(\frac{t_{s}}{t}\right)}^{2}+3\frac{t_{f}}{t}\frac{t_{s}}{t}\right]}{E_{f}\frac{t_{f}}{t}+E_{s}\frac{t_{s}}{t}},$$

(2c) $$\rho_{eff}=\frac{\rho_{s}\times t_{s}+\rho_{f}\times t_{f}}{t_{s}+t_{f}},$$

where *ρ* is the mass density [kg/m^3^]. It is observed that any attempt to increase the deflections will decrease the resonant frequency. The deflection and natural frequency contours shown in Figure 2 are plotted based on Equations (1) and (2), respectively. It is evident that, for any given thickness, the deflection increases with the increase in length of the simplified microcantilever. In addition, the optimum design space is given as the shaded area in Figure 2. Therefore, the geometry of the microcantilever tested in this study was selected from within this region.

### 2.2. Thermal Bimorph Microcantilevers

**Figure 3:**
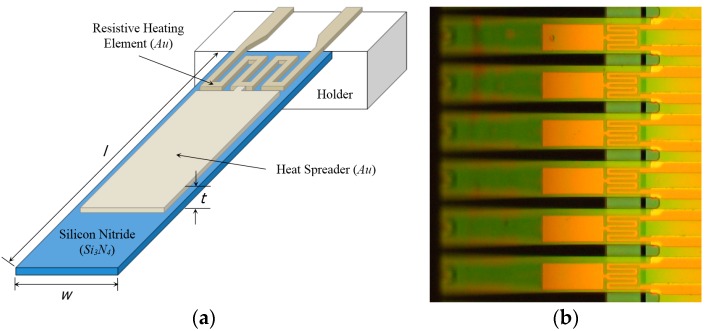
The bimetallic microcantilever used in this study: (**a**) A Schematic design; (**b**) Optical image of fabricated microcantilevers.

The microcantilevers used in this study were fabricated based on the commercial foundry process (courtesy of NanoINK, Inc., Campbell, CA, USA) [2]. Figure 3 presents a schematic of a typical bimorph microcantilever and defines the geometry. The length and thickness of the base material of the microcantilever (i.e., silicon nitride) was selected based on the sensitivity analysis and design optimization results shown in Figure 2. In addition, it was designed such that the Au layer has two different sections for resistive heating and heat spreading. Most importantly, the geometry of the resistive heating element was designed to be advantageous for Joule heating. Further, the coverage of the thin Au film over the Si_3_N_4_ microcantilever is determined according to heat flux required for the optimum deflection range. The microcantilever has nominal dimensions of 150 µm in length (*l*), 30 µm in width (*w*), and 1 µm in thickness (*t*). Bilayer structures consist of two layers of materials: a Si_3_N_4_ substrate of 600 nm thickness with a 400 nm thick deposited Au layer on top. For the substrate thickness of 600 nm, the optimized length of the microcantilevers can be selected in the region from 120 to 160 nm, which is consistent with the design presented in this study. The micro-fabricated microcantilever is connected to a flexible printed circuit board (PCB) for electrical actuation.

Microfabrication based on photolithography is excellent for achieving small absolute tolerances as opposed to material removal by machining. Nevertheless, the substantial geometrical variations for microcantilevers fabricated with MEMS processing technologies (e.g., photolithography) can significantly affect the performance of the sensor. The manufacturing tolerance was analyzed as the values of ±2 µm in length and ±10% in heater size, respectively. The variations due to manufacturing tolerance can affect the absolute values of deflections, which results in differences with the simulation results. This is because the nominal dimensions were used for numerical modeling in this study.

**Figure 4:**
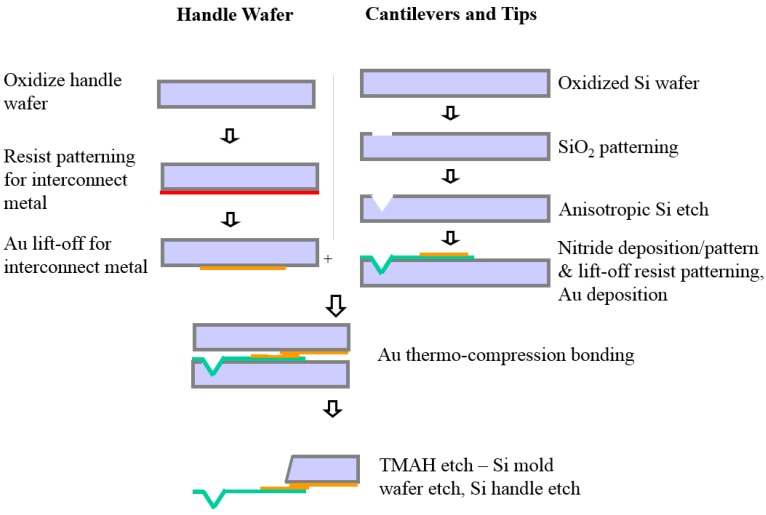
Cross-sectional view of the microcantilever fabrication scheme.

As shown in Figure 4, the bilayer structure is fabricated by thermal compression bonding at high temperatures (~350 °C) of a Au film deposited on a Si wafer by evaporation at low pressure (2–7 × 10^−7^ torr), and the Si_3_N_4_ microcantilever was made by etching and metallization on an oxidized Si wafer. In the process of thermal bonding between two different metal layers, the thermal stresses (or residual stresses) are induced. This causes an irreversible initial deflection at room temperature. This factor should be considered in the design of a microcantilever-based sensor system using the optical deflection method, as it can affect the initial position of the reflected light spot.

## 3. Theoretical Modeling of Bending Characteristics

### 3.1. Residual Stress

**Figure 5:**
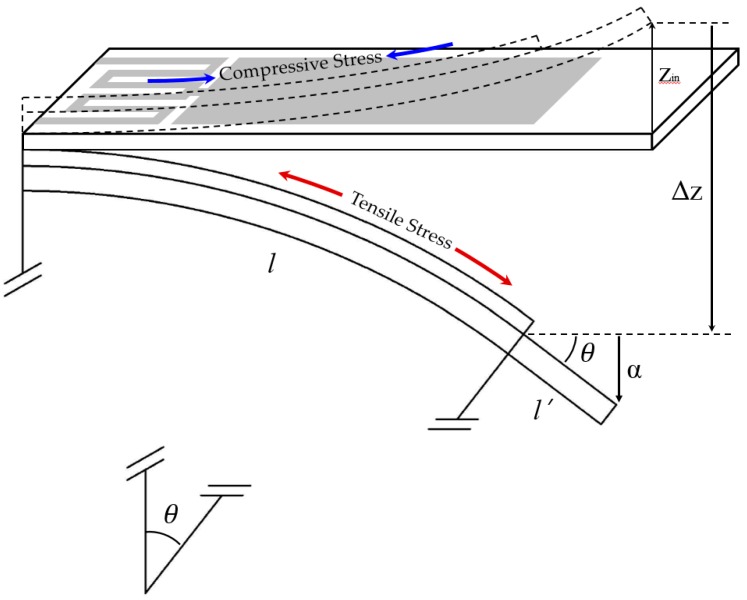
Geometrical representation of bending characteristics of thermal bimorph microcantilevers for theoretical calculation.

Metal films can be deposited by sputtering, evaporation, electroplating, and chemical vapor deposition (CVD). During the deposition processes, the substrate and film undergo heating and cooling cycles. Recrystallization and grain growth occurring at temperatures exceeding the elastic limit cause an irreversible deformation, which results in the initial deflection, e.g., *z_in_* in Figure 5 [25]. In this case, differential stress (also called residual stress) is created due to the dissimilar CTEs of the Si_3_N_4_ substrate and Au film. Residual stress in multilayer systems is a important issue, because it sometimes results in cracking or interfacial failure [25]. Considerable effort has been devoted to analyzing these residual stresses [13,20,21,25]. The first attempt to relate the residual film stress to the curvature of a bilayer film/substrate system was performed by Stoney [13], and the equation he developed has been adopted extensively [20]. However, in these studies, it is assumed that the film thickness is infinitesimal (or $t_{Au}<<t_{Si_{3}N_{4}}$) compared to the substrate thickness. In cases where the stress distribution through the thickness is not significant, the average residual film stress is calculated by [19].

(3) $${\bar{\sigma }}_{f}=\frac{1}{t_{f}}\int_{0}^{t_{f}}\sigma_{f}dz=\frac{E_{s}E_{f}t_{s}\left(E_{s}t_{s}{}^{3}+E_{f}t_{f}{}^{3}\right)\left(\alpha_{s}-\alpha_{f}\right)\Delta T}{E_{s}^{2}t_{s}^{4}+E_{f}^{2}t_{f}^{4}+2E_{s}E_{f}t_{s}t_{f}\left(2t_{s}^{2}+2t_{f}^{2}+3t_{s}t_{f}\right)},$$

where $\sigma_{f}$ is defined as the mismatch between the elastic stress *Eε* and the thermal stress *α*Δ*T*.

### 3.2. Electro-Thermo-Mechanical Modeling

Various theoretical approaches for the strain induced by thermal actuation have already been investigated [22,23,24]. In most theoretical models, the effects of thickness and thermal resistance between layers are neglected, and the strains at the interface of each adjacent layer are assumed to be equal. In this study, to directly compare the results according to the applied current, we adopt the electro-thermo-mechanical coupling model proposed by Jiang et al. [24]. However, the Au film of the bimorph microcantilever proposed in this study has relatively complex geometry, as shown in Figure 2a. Therefore, the total deflections of microcantilevers are determined by including additional deflections from the extension of single crystal structures (*l’*) without deposition of an Au film. This is because the bimetallic effect need not be considered for this extended region. The geometrical representation of resultant deflections (Δz_tot_) is shown in Figure 5, and their mathematical definition is expressed as follows:

(4a) $$\begin{array}{ll} \Delta z_{tot}= & \frac{DA^{-1}C}{2-DA^{-1}B}\;\times \;\frac{4l^{4}I^{2}R}{\sum_{i=1}^{2}K_{i}V_{i}\mu_{1}^{4}\sinh\left(\mu_{1}\right)}\\ & \;\times \left[\mu_{1}^{2}\sinh\left(\mu_{1}\right)-16\sinh\left(\frac{\mu_{1}}{2}\right)+4\mu_{1}-4\mu_{1}\cosh\left(\mu_{1}\right)+8\sinh\left(\mu_{1}\right)\right]+l^{\prime }\sin\left[\tan^{-1}\left(\frac{\Delta z}{l}\right)\right] \end{array},$$

with

(4b) $$A=\left[\begin{array}{cc} {\left(E_{1}A_{1}\right)}^{-1} & {\left(E_{2}A_{2}\right)}^{-1}\\ 1 & 1 \end{array}\right],\;B={\left[\begin{array}{cc} t_{1} & t_{2} \end{array}\right]}^{T},\;C={\left[\begin{array}{cc} \alpha_{2}-\alpha_{1} & 0 \end{array}\right]}^{T}\;\mathrm{and}\;D=\frac{\left[\begin{array}{cc} t_{1}/2 & \left(t_{1}+t_{2}\right)/2 \end{array}\right]}{{\left(EI\right)}_{eq}},$$

(4c) $$\beta_{0}=\sqrt{\frac{1/a_{1}+1/a_{2}}{B_{0}}}\;\mathrm{where}\;a_{i}=\frac{\sum_{i=1}^{2}K_{i}V_{i}}{\left(V_{1}+V_{2}\right)h_{i}},$$

(4d) $$\mu_{1}=2l\sqrt{\beta_{0}^{2}+\frac{hS}{\sum_{i=1}^{2}K_{i}V_{i}}},$$

where *I* is the applied current, *R* is the resistance of heating element, *B*_0_ is the layer thickness, *(EI)_eq_* is the equivalent flexural rigidity, *h* is the convective heat transfer coefficient, *V_i_* is the volume of the *i*th layer, *K_i_* is the thermal conductivity of the *i*th layer, and *S* is the surface area.

## 4. Numerical Modeling of Bending Characteristics

Numerical techniques, such as finite differential methods (FDM) or finite element methods (FEM), are normally used for structural dynamics simulations. In this study, an electric-thermo-structural coupling simulation is required for calculation of the change in surface stresses resulting from heat conduction induced by electrical actuation. The volumetric Joule heat generated by an electric current through a resistive heating element can be calculated from Ohm’s law, as follows:

(5) $$Q=I^{2}R=I^{2}\rho \frac{l}{A}\to q=\frac{Q}{V}={\left(\frac{I}{A}\right)}^{2}\rho ,$$

In particular, proper estimation of the temperature profile of the microcantilevers is a key factor affecting the prediction of the mechanical deflection with changes in the temperature distribution of the bimorph structure. For numerical simulations, ANSYS Mechanical APDL and ESI CFD-ACE+ were used to calculate the deflection, based on FEM. The simulations were performed on three-dimensional FE models of the cantilevers under linear and static conditions.

**Figure 6:**
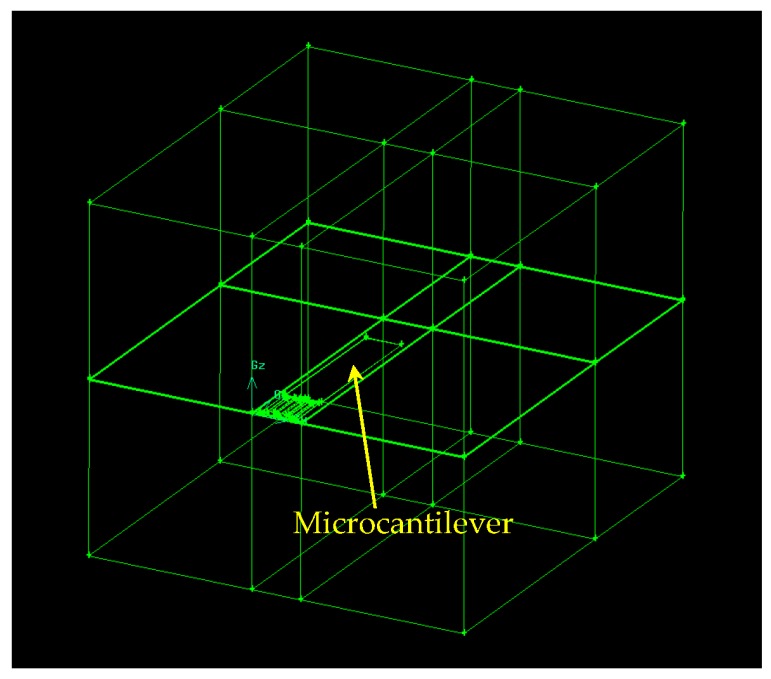
Geometry of the control volume for simulation (it is initially assumed to be filled with air).

The temperature profile of the microcantilever resulting from a conductive and convective heat transfer analysis is achieved with a computational fluid dynamics (CFD) tool (Fluent), which serves as the initial condition for the structural dynamics simulation using ANSYS Mechanical APDL. CFD simulations were performed based on the 3-D, laminar, species transport, and steady-state simulation techniques. Hexagonal and gradient meshing techniques were used. Figure 6 shows the solid model generated in Gambit software for thermal analysis using Fluent.

**Figure 7:**
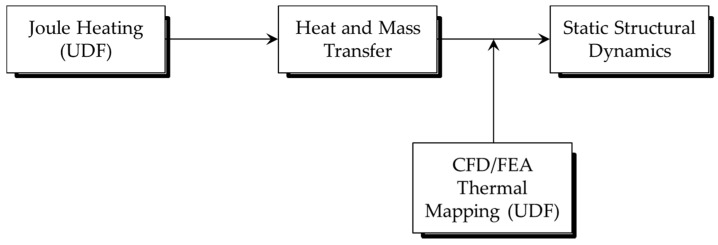
Schematic of a complete numerical model for electro-thermo-mechanical behavior analysis using ANSYS.

The schematic procedure of the numerical simulations is shown in Figure 7. The deflection due to residual stress can be initially applied before the main calculations. Deflection is calculated by a static-structural simulation based on thermal data obtained from the results of an electric-thermal coupling simulation. To calculate the mechanical deflection caused by the thermal stress at the surface of the microcantilevers, UDF (User-Defined Code) code for CFD/FEA thermal mapping was implemented in the Fluent calculation. The limitation of this approach is that the meshing and scaling of the models needs to be consistent in both Fluent and ANSYS Mechanical APDL. On the other hand, ESI CFD-ACE+ offers a more straightforward environment to model multiphysics systems. Heat transfer, stress, grid deformation, and electric modules were selected for this case, and geometrical and material properties were identical to the case in ANSYS.

**Figure 8:**
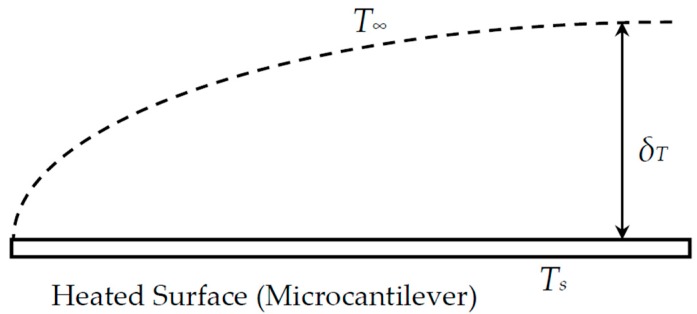
Schematic representation for thermal boundary layer on natural convection over a horizontal plate.

In general, the material properties of metallic films are different from those of the bulk. The material properties used in this study are summarized in Table 1. The heat supplied by the resistive heater is treated as a function of temperature, due to the temperature-dependent resistivity of the gold heating element. To determine the control volume for simulation, the thickness of the thermal boundary layer (***δ_T_***) on natural convection over a heated horizontal plate shown in Figure 8 is calculated by the following equation [26].

(6) $$\delta_{T}=0.0014{\left[\frac{g\beta }{\nu \alpha }\left(T_{s}-T_{\infty }\right)L^{3}\right]}^{0.24}$$

where *g* is the acceleration due to gravity (9.81 m/s^2^), *β* is the thermal expansion coefficient [K^−1^], *T_s_* is the wall temperature [K], *T_∞_* is the ambient temperature [K], *ν* is the kinematic viscosity [m^2^/s], *α* is the thermal diffusivity, and *L* (= Area/Perimeter) is the characteristic length [m]. For the size of the simulation volume determined from the calculation using Equation (6), the convective heat transfer coefficient (*h*) calculated based on the pure conduction correlation by the Nusselt number (i.e., Nu = 1) was consistent with the value obtained from the experiments (*h* = 700 W/m^2^·K) [2,27]. Because the actuation slew rate was less than 10 μs and the actuation latency was less than 5 ms, we selected 4 ms as the total simulation time for a steady-state simulation.

## 5. Experimental Measurements

**Figure 9:**
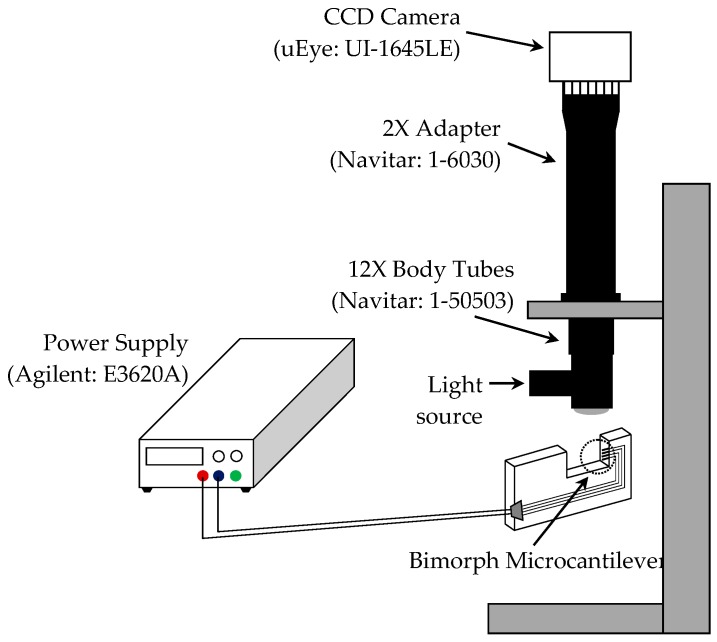
Schematic setup of measurement experiment.

Generally, the change in vertical position of microcantilevers can be monitored by tracking the reflected light spot on a projection screen or by using position sensitive detectors (PSD) [2,3,4,5,24]. This method requires a high-resolution PSD and the precise value of the distance between the end of the cantilever and the detector, to determine the actual vertical movement of the microcantilever tip from the variations in positions of the reflected light spot. Therefore, in this study, we adopted a method of visually capturing the dynamic motion caused by thermal actuation resulting from electrical current using an optical system. Figure 9 shows a schematic illustration of the experimental setup for measuring the deflection of microcantilevers. The deflection is measured by comparing the locations of the end of the microcantilevers. The combination of a CCD camera (purchased from IDSTM, Model: uEye UI-1645LE) and lenses (purchased from Navitar, Model: 12X UltraZoom and 2X adapter) enabled us to obtain images with resolution of ~ 0.7 μm per pixel. Ultimately, this makes it possible to measure the deflection of the microcantilever by measuring the pixel sizes using a commercial image processing tool (e.g., Photoshop^TM^).

## 6. Results and Discussion

**Figure 10:**
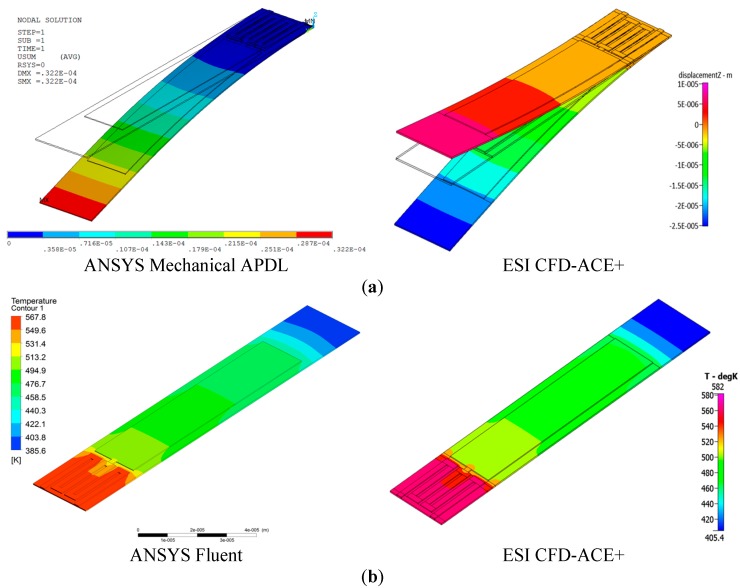
(**a**) Deflection of bimorph microcantilever that occurred by thermal actuation. Total deflection (ANSYS: 32.2 μm/ESI CFD-ACE+: 35.45 μm) is determined by the sum of deflections by residual stress and thermal actuation and (**b**) temperature profile of the thermally actuated bimorph microcantilever at the applied current of 20 mA.

The effect of residual stress from heat treatment in the fabrication process is usually characterized by the geometrical factors, material properties, and, most importantly, temperature variations during heat treatment [19,21]. In this study, we analyzed only the thermally induced stress, and excluded intrinsic stress resulting from defects and impurities incorporated in the material. Because deflection due to residual stress is generated when metal layers with different thermal expansion coefficients are thermally bonded, for the application of the theoretical model, we considered only the length corresponding to the area where the Au thin film was deposited. Thermal deformation exceeding the elastic limit cannot be completely recovered to the original status, even at room temperature. The estimation of initial deflection by residual stress is shown in Figure 10a. The average residual film stress calculated by Equation (3) was approximately −0.11 GPa. As seen in the results from ESI CFD-ACE+, shown in Figure 10a, the negative stress causes the microcantilever to be inversely deflected. On the other hand, the results from ANSYS Mechanical APDL were represented as the final position, including the initial deflection by residual stress. After calculating the initial deflection (i.e., approximately 10 μm), we were able to subsequently obtain the final positions of the end of the electrically actuated microcantilever from electro-thermal-structural coupling simulation techniques. Figure 10a shows the resultant deflections of the microcantilever. Because the deflection by thermal actuation occurred in the inverse direction of deflection due to residual stress, the total deflected distance can be defined as the sum of the deflections caused by residual stress and thermal actuation.

Heat is generated by the Au thin-film resistive heating element by actuating current. These heat flows are conducted to the entire region of the microcantilever through the thin-film Au heat spreader. As shown in Figure 10b, the conducted heat flows in the microcantilevers heated up to high temperatures; for instance, the actuation current of 20 mA heated it to 580 K. Further, we can see that the temperature distribution of the electrically heated microcantilever was not uniform. This was due to convective heat transfer by the high heat transfer coefficient (*h* = 700 W/m^2^·K) in micro-scale thin film materials. Moreover, the theoretical model did not reflect the temperature dependence of the resistivity of the Au heating element. The nominal resistance value for application of the theoretical model given by Equation (4) is 21 Ω, which was the value measured using a multimeter. Figure 10a represents the thermally induced deflection due to the bimetallic effect, which is simulated based on the thermal conditions shown in Figure 10b.

**Figure 11:**
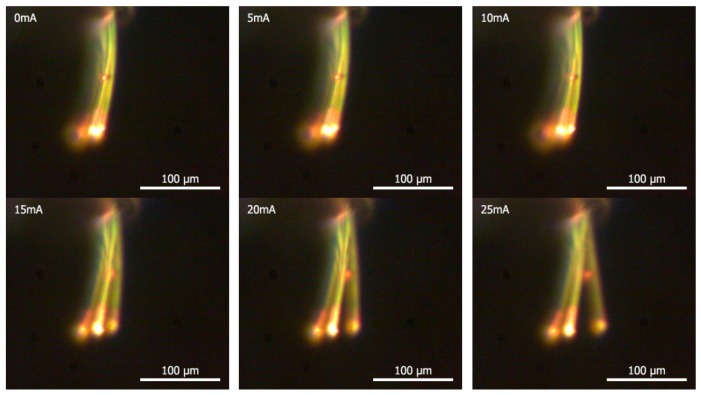
Flexural motions of bimorph microcantilevers thermally activated by electrical current (0–25 mA).

**Figure 12:**
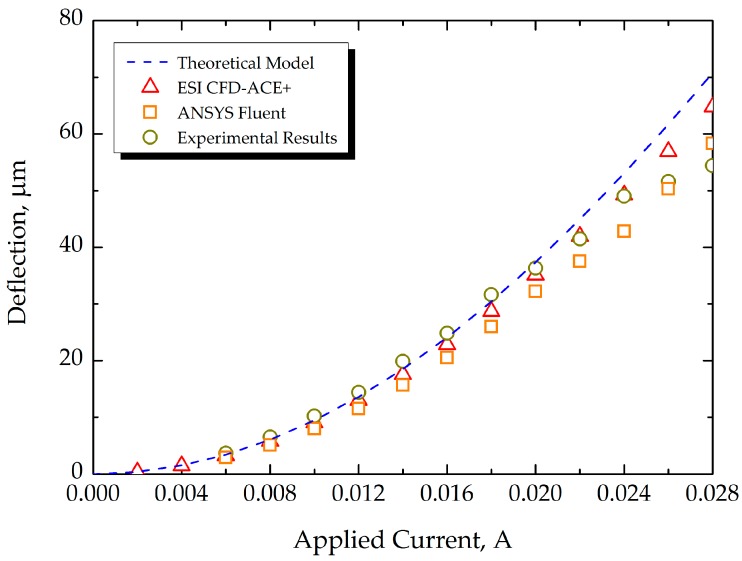
Comparison of deflection among FEA models (ANSYS and ESI CFD-ACE+), theoretical model, and experimental data.

**Figure 13:**
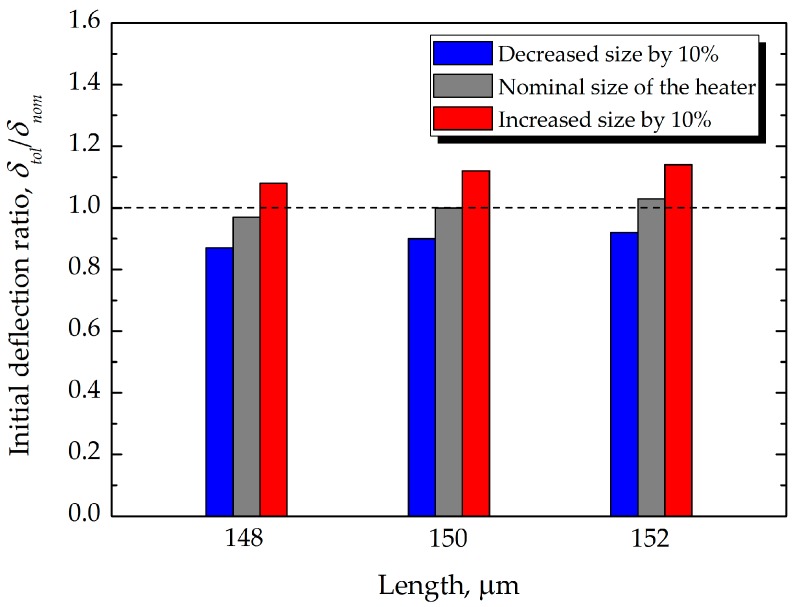
Comparison between changes in initial deflection ratio resulting from manufacturing tolerance.

To ascertain the validity of the FEA of microcantilever deflection by thermal actuation, the FEA results are plotted together with the results of the analytical model given in Equation (4) and our experimental data (as shown in Figure 11), as shown in Figure 12. The flexural motions of bimorph microcantilevers, shown in Figure 11, were measured using a CCD camera while the microcantilevers were electro-thermally actuated by an electric current from a power supply, as shown in Figure 9. In Figure 12, the deflection due to the theoretical model is plotted when using geometrical and material properties of the microcantilever defined in this study. In the region of ~25 mA, all of the models and the experiment show good agreement, having errors of 10%, on average. The simulation results of two commercial multi-physics tools showed a similar trend up to 20 mA, and the results by ESI CFD-ACE+ showed a relatively closer relation to experiments in the region of operating actuation currents. However, at actuation currents exceeding 25 mA, nonlinear behavior characteristics were observed in the experimental results, which was more pronounced in the numerical results of ANSYS than those of ESI CFD-ACE+. It was determined that this disagreement resulted from both the radiation effect between microcantilevers in an array and the nonlinear behaviors of overheated microstructures. Because the analytical and numerical models follow linear characteristics, they cannot completely account for the nonlinear behaviors of overheated microstructures. Nevertheless, it was clearly demonstrated that the decrease in the deflections at currents greater than 25 mA, compared to the theoretical model, resulted from the reduced heat transfer rate from the heating element to the heat spreader due to a high convective heat transfer rate (i.e., *h* = 700 W/m^2^·K) to the air. In addition, the deflection characteristics dependent on variations of length and heat size were numerically evaluated to investigate the effect of manufacturing tolerance on the bending response of the microcantilevers. From the results, the deflection ratio between the nominal dimension (*δ_nom_*) and changes (*δ_tol_*) by manufacturing tolerance was calculated to be within the range of 0.87–1.14, as shown in Figure 13.

## 7. Summary and Conclusions

A theoretical and numerical approach for investigating the bending behavior of a bimetallic microcantilever based on the multi-physics modeling method and its corresponding experimental validation using optical measurements have been presented in this study. In the region of optimum current (or valid operational current) to prevent deterioration by overheating, the theoretical model, and the FEA results showed very good agreement (errors of 10%, on average) with the experiments. The simulation results were very sensitive to the material properties, and thus it was necessary to find the appropriate values for a thin-film metal, rather than the bulk properties. In addition, the heat transfer characteristics at the micro/nanoscale (e.g., estimating the equivalent convective heat transfer coefficient due to pure conduction during thermal actuation of MEMS devices) should be properly considered to enhance the accuracy of numerical prediction. The final deflection due to thermal actuation occurs when the heat loss by convection from the heated surface is balanced by the heat generation by Joule heating. The impact of the design parameters (e.g., dimensions, thermo-physical properties, actuation currents, manufacturing tolerance, and so on) on the electro-thermal actuation and mechanical-response of the microcantilever sensor was successfully assessed in this study. These results are essential precursors for the prediction of mass or temperature variations by adsorption or chemical reactions of an analyte on the surface of a pre-heated microcantilever. In addition, the procedure of this study offers a guideline for the design optimization of microcantilevers using different materials and dimensions.

## Figures and Tables

**Table 1:** Material properties used in this study (data were found from studies on thin metal films).

Property	Si_3_N_4_	Au
Thermal Conductivity (k), [W/m·K]	1.7 [27]	150 [28]
Thermal Expansion Coefficient (*α*), [K^−1^]	0.3 × 10^−6^ [27]	14.6 × 10^−6^ [29]
Elastic Properties *E*: Young’s Modulus [GPa]/*ν*: Poisson’s Ratio	224.6 [27]/0.253 [30]	74.5 [27]/0.35 [31]
Electrical Resistivity (*R*), [Ωm]	1 × 10^10^ [32]	2.214 × 10^−8^ [33]
